# The complete mitochondrial genome of *Potomida acarnanica* (Kobelt, 1879)

**DOI:** 10.1080/23802359.2024.2353271

**Published:** 2024-06-03

**Authors:** Ana Matos, André Gomes-dos-Santos, Amílcar Teixeira, Simone Varandas, Ronaldo Sousa, Ioannis Karaouzas, Stamatis Zogaris, Elsa Froufe, Manuel Lopes-Lima

**Affiliations:** a CIIMAR/CIMAR—Interdisciplinary Centre of Marine and Environmental Research, University of Porto, Matosinhos, Portugal; b Centro de Investigação de Montanha (CIMO), Instituto Politécnico de Bragança, Bragança, Portugal; c Mountain Research Centre, School of Agriculture, Polytechnic Institute of Bragança, Bragança, Portugal; d Forestry Department, Centre for Research and Technology of Agro-Environment and Biological Sciences, University of Trás-os-Montes and Alto Douro, Vila Real, Portugal; e CBMA—Centre of Molecular and Environmental Biology, Department of Biology, University of Minho, Braga, Portugal; f Hellenic Centre for Marine Research, Institute of Marine Biological Resources and Inland Waters, Anavyssos, Greece; g CIBIO, Centro de Investigação em Biodiversidade e Recursos Genéticos, InBIO Laboratório Associado, Universidade do Porto, Vairão, Portugal; h BIOPOLIS Program in Genomics, Biodiversity and Land Planning, CIBIO, Vairão, Portugal

**Keywords:** Mitogenome, freshwater mussels, phylogeny

## Abstract

Freshwater mussels (Bivalvia, Unionida) play essential roles in the well-functioning of ecosystems, even providing essential services to humans. However, these bivalves face numerous threats (e.g. habitat loss and fragmentation, pollution, introduction of invasive species, and climate change) which have already led to the extinction of many populations. This underscores the need to fully characterize the biology of these species, particularly those, such as *Potomida acarnanica*, that are still poorly studied. This study presents the first mitogenome of *P. acarnanica* (Kobelt, 1879), an endemic species of Greece with a distribution limited to only two river basins. The mitochondrial genome of a *P. acarnanica* specimen, collected at Pamisos River (Peloponnese, Greece), was sequenced by Illumina high-throughput sequencing. This mitogenome (16,101 bp) is characterized by 13 protein-coding genes, 22 transfer RNA and 2 ribosomal RNA genes. The size of this mitogenome is within the range of another *Potomida* mitogenome already published for the species *Potomida littoralis*. In the phylogenetic inference, *P. acarnanica* was recovered as monophyletic with *P. littoralis* mitogenome in the Lamprotulini tribe, as expected. This genomic resource will assist in genetically characterizing the species, potentially benefiting future evolutionary studies and conservation efforts.

## Introduction

Freshwater mussels play a crucial role in maintaining the health and balance of aquatic ecosystems (Strayer 2014). These mollusks contribute to nutrient cycling and act as nature’s water purifiers, filtering and cleansing the water they inhabit, helping to sustain a diverse array of aquatic life and supporting the overall health of freshwater ecosystems (Lopes-Lima et al. 2017; Vaughn 2018). Additionally, they also hold cultural and economic importance, as they have been used historically for food, tools, and even as sources of pearls (Strayer 2017; Zieritz et al. 2022). Nevertheless, freshwater mussel populations face numerous threats, such as habitat loss and fragmentation, pollution, introduction of invasive species, and climate change, underscoring the urgency of conservation efforts to safeguard these vital organisms and the ecosystems they inhabit (Ferreira-Rodríguez et al. 2019; Lopes-Lima et al. 2023). In fact, several freshwater mussel species, especially those that are less abundant and have restricted ranges, are now extinct or highly threatened (Lopes-Lima et al. 2018).

In the Mediterranean region, where freshwater mussel species endemism is high, their habitats are especially affected by water scarcity with the climate crisis and water over-exploitation exacerbating the situation. The Greek endemic *Potomida acarnanica* (Kobelt, 1879) is restricted to only two Greek river basins, i.e., Pamisos and Acheloos, that are affected by several anthropogenic pressures (Froufe et al. 2016; Skoulikidis et al. 2022). Due to its limited distribution, this poorly known species is at high risk of extinction.

## Materials and methods

**Figure 1.**
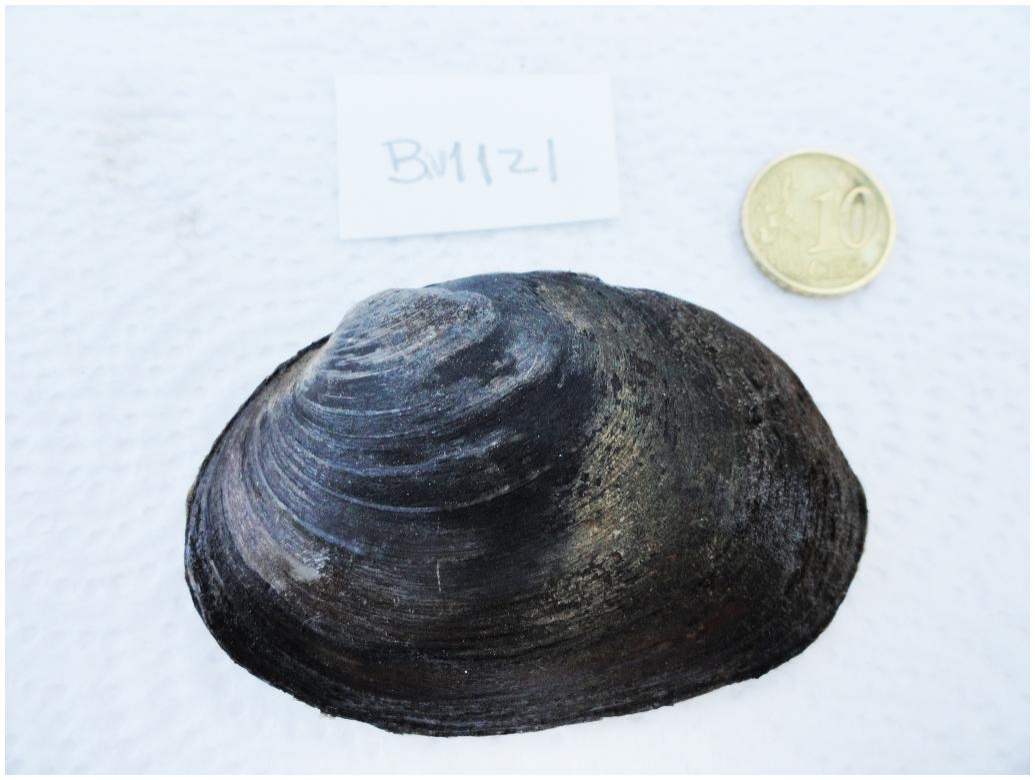
Species image reference of *P. acarnanica* (photograph by Manuel Lopes-Lima).

A specimen of *P. acarnanica* was collected on 27^th^ September 2014 at Pamisos River (Peloponnese, Greece) (37.123287, 21.990056) by Manuel Lopes-Lima (Figure 1). The voucher specimen has been deposited at the Museum of Natural History and Sciences of the University of Porto, Portugal (https://mhnc.up.pt/, Manuel Lopes-Lima, manuelpmlopeslima@gmail.com) with voucher name MHNC-UP BIV1121. Genomic DNA was extracted from foot tissue following a standard high-salt protocol (Sambrook et al. 1989). The extracted genomic DNA was sent to the Deakin Genomics Center (Melbourne, Australia) for Illumina Paired-End (PE) library construction (2x150bp) and whole genome sequencing using a MiSeq Illumina platform. Mitogenome assembly was obtained using NOVOPlasty (v.4.2) (Dierckxsens et al. 2017) and annotation was conducted using MITOS2 web server (Bernt et al. 2013, Al Arab et al. 2017, Donath et al. 2019) with default parameters. Burrows–Wheeler Aligner v.0.7.17-r1198 (Li 2013) was used to create the coverage plot by mapping the PE reads to the final assembly, the respective graphical plot was generated using bam2plot (https://github.com/willros/bam2plot) (Supplementary Figure 1). Seventeen mitogenome sequences (NC_015110.1, NC_011763.1, NC_044110.1, MW242814.1, MW242812.1, MW242816.1, MW242818.1, NC_023250.1, NC_022701.1, NC_044111.1, NC_039839.1, NC_044112.1, NC_044124.1, NC_030073.1, AB055625.1, NC_023346.1, NC_030336.1) from the Unionidae family (Gonideinae subfamily), were retrieved from GenBank (22^nd^ December 2023). Moreover, two mitochondrial genomes (from *Amblema plicata* (NC_050056.1) and *Margaritifera* (NC_043836.1)) were downloaded from Genbank (22^nd^ December 2023) as outgroup. The 13 protein-coding genes of the downloaded mitochondrial genomes were aligned, trimmed and concatenated with MAFFT (default parameters) (version 7.505) (Katoh and Standley 2013), trimAL (version 1.2) (-gt 0.5) (Capella-Gutiérrez et al. 2009) and FasConCAT-G (-p -p -a -s -l) (version 1.05.1) (Kück and Longo 2014), respectively. The final alignment had 11148 bp. IQ-TREE (version 1.6.12) (-m TESTNEWMERGE -rcluster 10) (Nguyen et al. 2015; Kalyaanamoorthy et al. 2017) was used to identify the partition-scheme, best-fit nucleotide substitution models and Maximum Likelihood phylogeny. The evolutionary models applied were TPM3 + F + I + G4 (ATP6), TPM3u + F + I + G4 (ATP8), TN + F + I + G4 (COIII), TN + F + I + G4 (COII), TIM3 + F + R4 (COI and ND4L), TPM3u + F+R4 (Cytb, ND1 and ND2), K3Pu + F + I + G4 (ND3), GTR + F+R4 (ND4 and ND5), and TVM + F + I + G4 (ND6).

## Results

**Figure 2.**
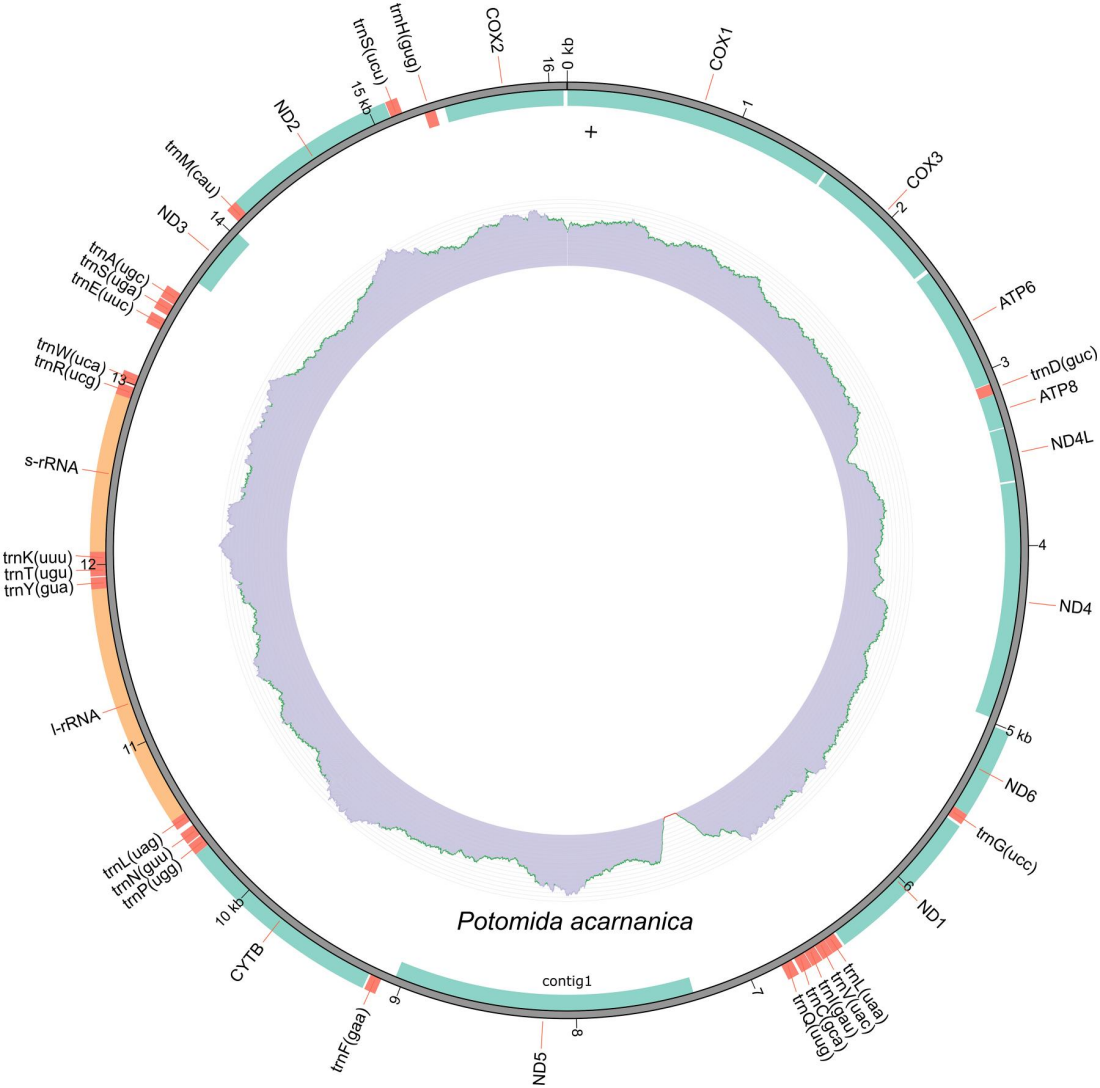
Mitogenome map of *P. acarnanica*. This plot was created with the annotation model of MITOZ. The outermost track displays the gene features and their strand positioning on the assembly. The color scheme are red for tRNA, green for PCGs, and orange for rRNAs. The Middle track represents read depth distribution across the assembly. The innermost track represents GC content distribution across the assembly.

The mitogenome of *P. acarnanica,* with a total of 16,101 bp, has 13 protein-coding genes, 22 transfer RNA (tRNA), and 2 ribosomal RNA (rRNA) genes (Figure 2). Twenty six of these genes are in the complementary strand (ND1 (NADH dehydrogenase subunit 1), ND2 (NADH dehydrogenase subunit 2), ND6 (NADH dehydrogenase subunit 6), cytochrome b (CYTB), 12S ribosomal RNA, 16S ribosomal RNA and 20 tRNA (tRNA^Gly^, tRNA^Leu^, tRNA^Val^, tRNA^Ile^, tRNA^Cys^, tRNA^Gln^, tRNA^Phe^, tRN^Pro^, tRNA^Asn^, tRNA^Leu^, tRNA^Tyr^, tRNA^Thr^, tRNA^Lys^, tRNA^Arg^, tRNA^Trp^, tRNA^Glu^, tRNA^Ser^, tRN^Ala^, tRNA^Met^ and tRNA^Ser^). This mitochondrial genome has been deposited in Genbank with accession number PP035751.

**Figure 3.**
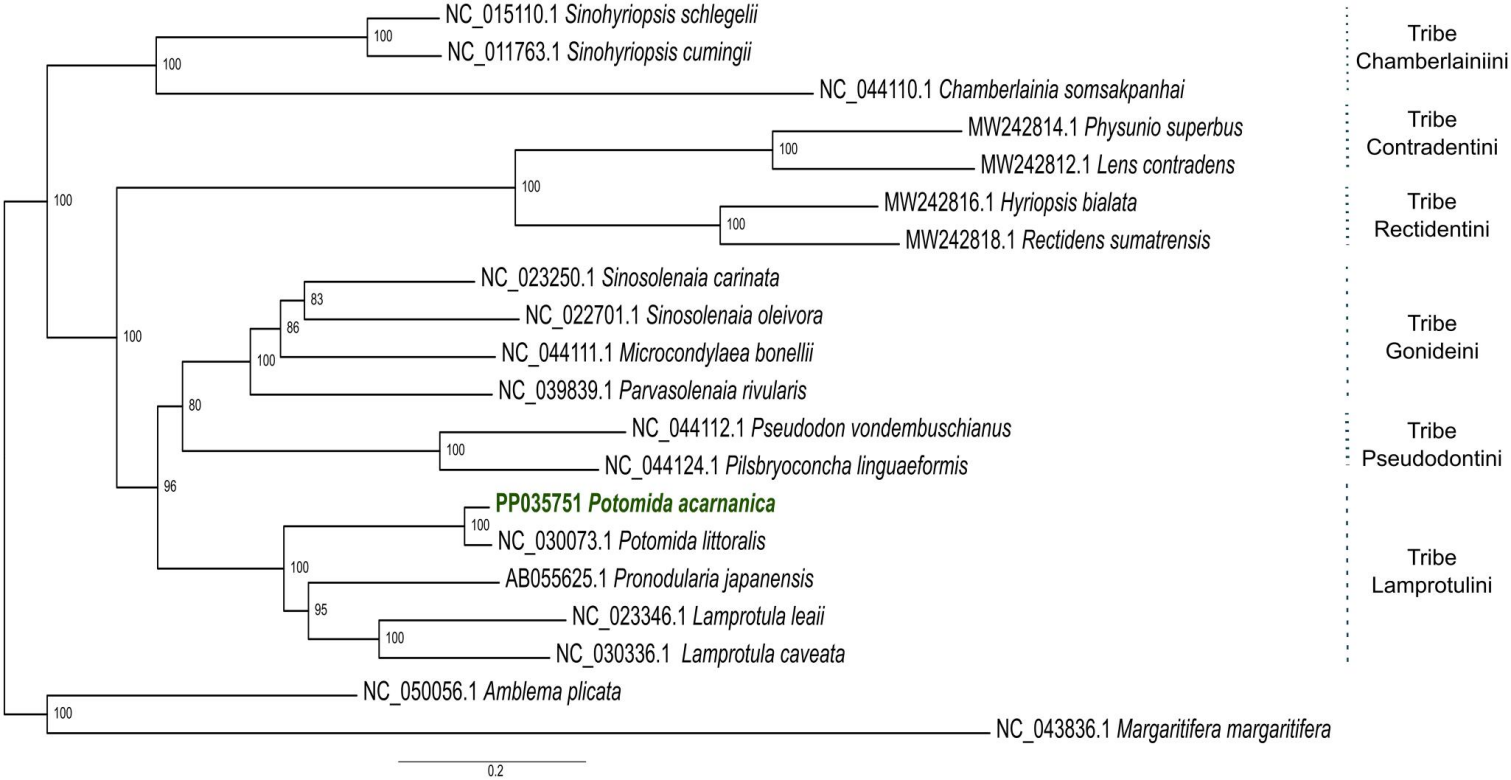
Maximum Likelihood Phylogenetic inference with all the downloaded mitogenomes (*n* = 19) and with the new mitochondrial genome of *P. acarnanica* (this mitogenome has been deposited in Genbank with accession number PP035751). The mitogenomes used in this phylogeny were: *Sinohyriopsis schlegelii* (NC_015110.1) (Sheng et al. 2014), *Sinohyriopsis cumingii* (NC_011763.1) (unpublished), *Chamberlainia somsakpanhai* (NC_044110.1) (Froufe et al. 2020), *Physunio superbus* (MW242814.1) (Zieritz et al. 2021), *Lens contradens* (MW242812.1) (Zieritz et al. 2021), *Hyriopsis bialata* (MW242816.1) (Zieritz et al. 2021), *Rectidens sumatrensis* (MW242818.1) (Zieritz et al. 2021), *Sinosolenaia carinata* (NC_023250.1) (Huang et al. 2013), *Sinosolenaia oleivora* (NC_022701.1) (Huang et al. 2015), *Microcondylaea bonellii* (NC_044111.1) (Froufe et al. 2020), *Parvasolenaia rivularis* (NC_039839.1) (unpublished), *Pseudodon vondembuschianus* (NC_044112.1) (Froufe et al. 2020), *Pilsbryoconcha linguaeformis* (NC_044124.1) (Froufe et al. 2020)*, Potomida littoralis* (NC_030073.1) (Froufe et al. 2016), *Pronodularia japanensis* (AB055625.1) (unpublished), *Lamprotula leaii* (NC_023346.1) (unpublished) and *Lamprotula caveata* (NC_030336.1) (unpublished). The two outgroup taxa used were: *Amblema plicata* (NC_050056.1) (Teiga-Teixeira et al. 2020) and *M. margaritifera* (NC_043836.1) (Gomes-dos-Santos et al. 2019).

In the phylogeny here provided, 17 mitogenomes of the subfamily Gonideinae (Unionidae family) are divided according to six tribes with high support (Figure 3). *P. acarnanica* and *Potomida littoralis* are recovered as monophyletic and sister to *Lamprotula caveata*, *Lamprotula leaii* and *Pronodularia japanensis* (Figure 3), all these five species belong to the Lamprotulini tribe (Figure 3).

## Discussion and conclusions

To date, only two mitochondrial genomes of *Potomida* have been available: the male (M-type) and female (F-type) mitogenomes of *Potomida littoralis* (Froufe et al. 2016b). This is the first complete F-type mitochondrial genome of *P. acarnanica*. The sex was determined using histology and this mitogenome presents the same F-type gene arrangement of this particular subfamily of Unionidae (Froufe et al. 2020). The single published F-type mitogenome of *P. littoralis* was 15,789 bp in length, similar to the length of the one presented here for *P. acarnanica* (16,101 bp). As expected, in the provided phylogenetic inference (Figure 3), the mitogenome of *P. acarnanica* is grouped with that of *P. littoralis*. This phylogeny is congruent with other phylogenetic reconstructions of the Gonideinae subfamily (Froufe et al. 2020). M-type mitochondrial DNA has been independently evolving from the F-type since the origin of the Unionida order thus they will be far more divergent than any F-type mitogenome of any Unionidae species included in the phylogenetic analysis (Froufe et al. 2016b). Given this divergence, only F-type mitochondrial sequences were included in the phylogenetic reconstruction.

For the *Potomida* genus, there is still a lack of molecular data. *Potomida acarnanica* is a poorly studied freshwater mussel associated with a high risk of extinction. Genomic resources are essential in evolutive studies and conservation management strategies (Garrison et al. 2021). Therefore, we present the first mitogenome of *P. acarnanica*. This will aid in the comprehensive genetic profiling of the genus and the development of conservation measures for this species.

## Supplementary Material

Supplemental Material

Supplemental Material

## Data Availability

The genome sequence data that support the findings of this study are openly available in GenBank of NCBI at https://www.ncbi.nlm.nih.gov under the accession number PP035751. The associated BioProject, SRA, and Bio-Sample numbers are PRJNA1056157, SRR27332421 and SAMN39090842 respectively.
