# Supplementary material for: The complete mitochondrial genome of Potomida acarnanica (Kobelt, 1879)
Source: Mitochondrial DNA B Resour. 2024 Jun 3;9(6):696–700. doi: 10.1080/23802359.2024.2353271 (PMC11149565; doi:10.1080/23802359.2024.2353271)
Supplement: Supplemental Material [file TMDN_A_2353271_SM0521.docx]

**Supplementary Material**

The complete mitochondrial genome of *Potomida acarnanica* (Kobelt, 1879)

Ana Matos^1^, André Gomes-dos-Santos^1^, Amílcar Teixeira^2^, Simone Varandas^3^, Ronaldo Sousa^4^, Ioannis Karaouzas^5^, Stamatis Zogaris^5^, Elsa Froufe^1^, Manuel Lopes-Lima^6,7,*^

^1^CIIMAR/CIMAR - Interdisciplinary Centre of Marine and Environmental Research, University of Porto, Matosinhos, Portugal;

^2^Centro de Investigação de Montanha (CIMO), Instituto Politécnico de Bragança, Campus de Santa Apolónia, 5300-

253 Bragança, PortugalMountain Research Centre, School of Agriculture, Polytechnic Institute of Bragança,

Bragança, Portugal

^3^Forestry Department, Centre for Research and Technology of Agro-Environment and Biological Sciences, University of Trás-os-Montes and Alto Douro, Vila Real, Portugal

^4^CBMA - Centre of Molecular and Environmental Biology, Department of Biology, University of Minho, Braga, Portugal

^5^Hellenic Centre for Marine Research, Institute of Marine Biological Resources and Inland Waters, 46.7 km Athens-Sounio Av., Anavyssos, 19013, Greece.

^6^CIBIO, Centro de Investigação em Biodiversidade e Recursos Genéticos, InBIO Laboratório Associado, Campus de Vairão, Universidade do Porto, 4485-661 Vairão, Portugal

^7^BIOPOLIS Program in Genomics, Biodiversity and Land Planning, CIBIO, Campus de Vairão, 4485-661 Vairão, Portugal

* **Corresponding author:** Manuel Lopes-Lima - [manuelpmlopeslima@gmail.com](mailto:manuelpmlopeslima@gmail.com), CIBIO, Centro de Investigação em Biodiversidade e Recursos Genéticos (CIBIO), InBIO Laboratório Associado, Campus de Vairão, Universidade do Porto, 4485-661 Vairão, Portugal and BIOPOLIS Program in Genomics, Biodiversity and Land Planning, CIBIO, Campus de Vairão, 4485-661 Vairão, Portugal.


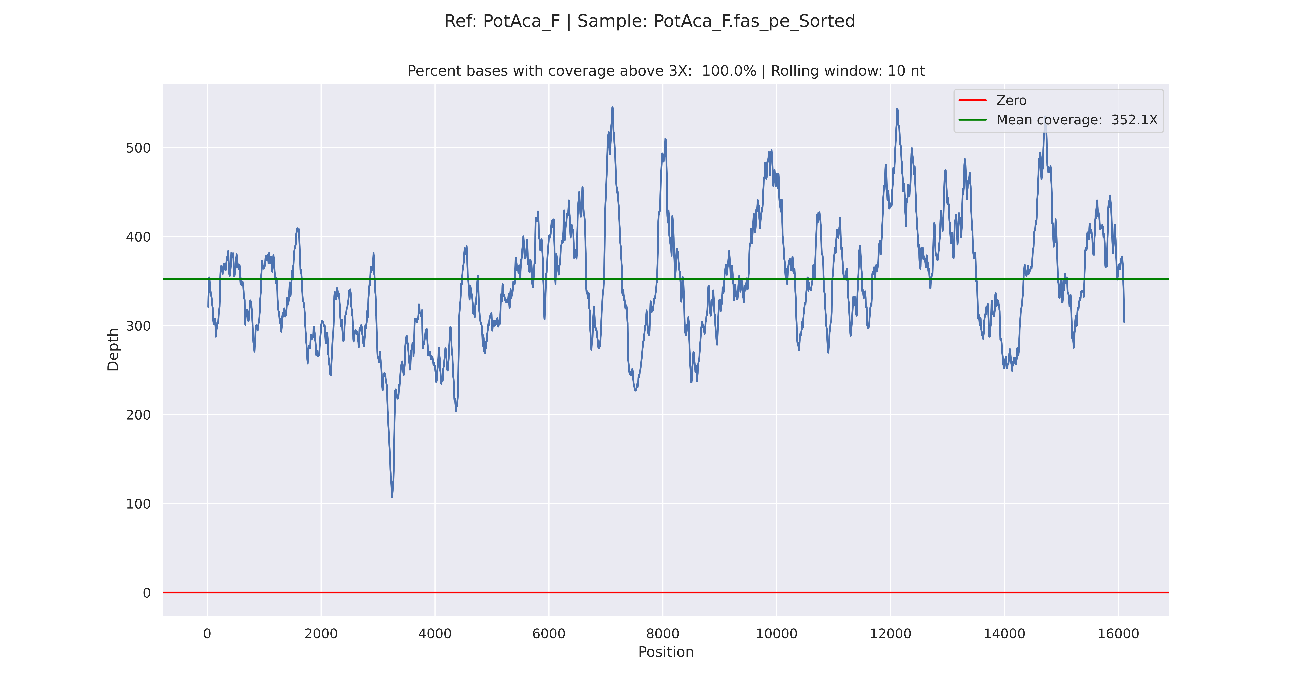
Supplementary Figure 1 - Read coverage plot of Potomida acarnanica mitogenome.
