# Supplementary figures and images for: The complete mitochondrial genome of Potomida acarnanica (Kobelt, 1879)
Source: Mitochondrial DNA B Resour. 2024 Jun 3;9(6):696–700. doi: 10.1080/23802359.2024.2353271 (PMC11149565; doi:10.1080/23802359.2024.2353271)

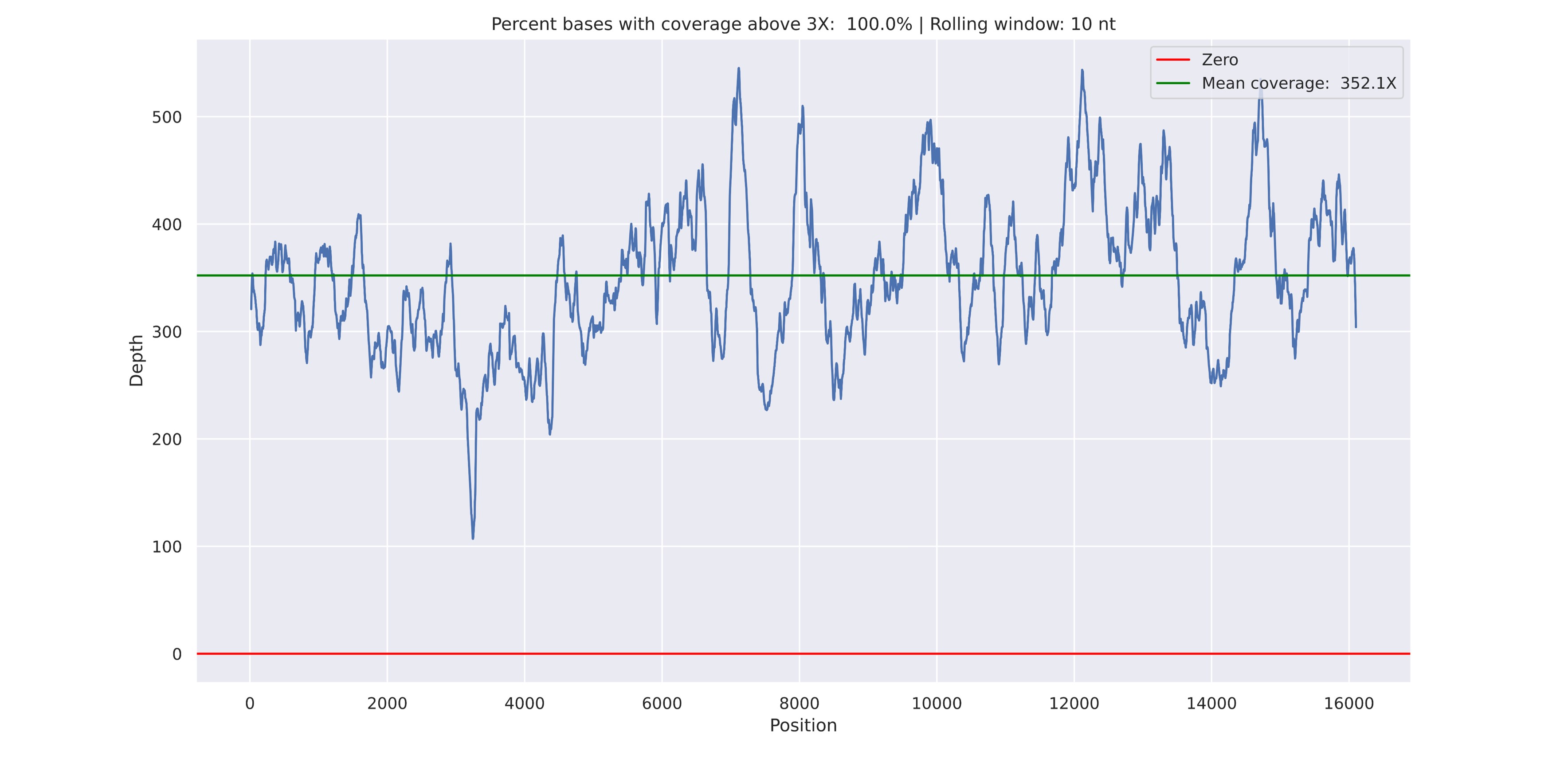

Supplement: Supplemental Material [file TMDN_A_2353271_SM0520.jpg]
